# Prospective randomized phase II study of FOLFIRI versus FOLFOX7 in advanced gastric adenocarcinoma: a Chinese Western Cooperative Gastrointestinal Oncology Group Study

**DOI:** 10.18632/oncotarget.18426

**Published:** 2017-06-09

**Authors:** Qiu Li, Feng Wen, Chengya Zhou, Meng Qiu, Jiyan Liu, Jing Chen, Cheng Yi, Zhiping Li, Deyun Luo, Feng Xu, Xiaohong Cai, Feng Bi, Western Cooperative Gastrointestinal Oncology Group of China

**Affiliations:** ^1^ Department of Medical Oncology, Laboratory of Signal Transduction and Molecular Targeting Therapy, Cancer Center, West China Hospital, Sichuan University, Chengdu, 610041, Sichuan Province, P.R. China; ^2^ Oncology Department, Sichuan Cancer Hospital, Chengdu, 610041, Sichuan Province, P.R. China

**Keywords:** gastric cancer, mFOLFIRI, mFOLFOX7

## Abstract

Until now, no standard chemotherapy has been widely accepted for advanced gastric cancer (GC). The current research aimed to compare folinic acid, fluorouracil with irinotecan (mFOLFIRI) or with oxaliplatin (mFOLFOX7) as first-line treatments in patients with locally advanced GC in an open, randomized, phase II study. Previously untreated metastatic or recurrent GC patients with measurable disease received mFOLFIRI (arm A) or mFOLFOX7 (arm B) every 2 weeks. The defined second-line treatment was mFOLFOX7 for arm A and mFOLFIRI for arm B. Primary endpoint was progression-free survival (PFS), and secondary endpoints were overall survival (OS), disease control rate (DCR) and toxicity. The evaluable population consisted of 128 patients (54 in arm A; 74 in arm B). Median PFS of arm A was 2.9 months (m) (95% confidence interval, *CI*, 1.9 to 4.1 m) versus 4.1 m (95% *CI*, 3.2 to 4.8 m) for arm B (*p* = 0.109). Median OS was 9.9 months (95% *CI*, 6.0 to 13.5 m) for arm A versus 12.0 m for arm B (95% *CI*, 10.3 to 13.7m; *p* = 0.431). DCRs for arm A and arm B were 59.3% and 66.3%, respectively (*p* = 0.850). In subgroup analysis of the patients who completed both treatment lines per protocol, the median first-line PFS was 2.1 m for the mFOLFIRI/mFOLFOX7arm versus 8.0 m for the mFOLFOX7/mFOLFIRI arm (*p* = 0.053), and the median second-line PFS values were 1.2 m versus 5.1 m (*p* = 0.287). Total PFS and OS were 8.1m and 11.0 m for the mFOLFIRI/mFOLFOX7 group compared with 12.2m and 20.2 m for the mFOLFOX7/mFOLFIRI group (*p* = 0.008, *p* = 0.030). Both regimens were well-tolerated with acceptable and manageable toxicities. Hence, there was no significant difference in the PFS or DCR. However, mFOLFOX7 followed by mFOLFIRI might have a better OS.

## INTRODUCTION

Although the incidence and mortality rates have declined worldwide, as the most common cancer, gastric cancer (GC) still ranks fourth in incidence and second in annual cancer-related deaths [1]. Approximately 70% of new cases and deaths occur in developing countries, and the number of Chinese GC patients(pts) accounts for 35–42% of cases worldwide [2, 3]. Generally, most pts have lost the surgery opportunity when first diagnosed. Even after surgery, the recurrence rate is relatively high. Additionally, the prognosis of advanced GC is poor, with a median survival of 3.0 to 5.0 months (m) if managed by best supportive care [4, 5].

For pts with advanced GC, palliative chemotherapy plays an important role in prolonging overall survival(OS) and improving the quality of life(QOL); however, until now, no standard chemotherapy has been widely accepted [5]. In recent years, chemotherapy regimens have been commonly used with a median survival of less than 10.0 months [6]. A well-recognized standard regimen for advanced or metastatic GC has not been established until now.

5-Fluorouracil (5-FU), as a primary chemotherapy drug against GC, is combined with leucovorin, resulting in a synergistic anti-tumor effect, which has been confirmed by many *in vivo* experiments [5, 6]. Currently, CF (cisplatin and 5-FU), widely used in North America, and ECF (epirubicin, cisplatin and 5-FU), commonly used in Europe, have become the basic treatments worldwide, and both regimens are regularly applied in China. Additionally, the prognosis of GC has improved with the new emergence of the third generation of anticancer drugs—for example, oxaliplatin and irinotecan.

In one of the largest clinical trials, REAL2, the new regimen efficacy of EOF (epirubicin, oxaliplatin and 5-FU) was not inferior to the original ECF in which cisplatin was replaced by oxaliplatin. Similarly, the effect of ECX (epirubicin, oxaliplatin and capecitabine), including capecitabine instead of 5-FU, was not inferior to the original ECF. Notably, the OS was significantly prolonged (median survival increasing from 9.9 m to 11.2 m) if cisplatin and 5-FU were substituted by oxaliplatin and capecitabine simultaneously in the so-called EOX regimen [7]. Additionally, another phase III clinical study, comparing the efficacy of FLO(5-FU, leucovorin, and oxaliplatin) and FLP(5-FU, leucovorin, and cisplatin), demonstrated the median PFS of FLO group enjoyed a longer trend but did not statistically meet significance for improvement (5.8 v 3.9 months, *P* = 0.077); however, in patients older than 65 years old, FLO was associated with a statistically prolonged PFS (6.0 v 3.1 months, *P* = 0.029) [8]. Therefore, oxaliplatin contained regimen have been the most widely option used in the first-line therapy of GC, including FOLFOX (5-FU, leucovorin, and oxaliplatin). FOLFOX is close but not exactly equivalent to FLO regimen, which does not contain bolus FU.

Irinotecan is a semi-synthetic anti-cancer drug derived from camptothecin. In 2006, a large-scale phase III clinical study named V306 suggested that irinotecan combined with 5-FU (IF) had a better tolerability and reactivity (32% vs 26%) than CF in North America, and the median survivals of IF and CF were 9.0 m and 8.7 m, respectively [9]. In addition, another phase II clinical study (136 pts) compared the efficacy among LF (fluorouracil and leucovorin), CLF (cisplatin, fluorouracil and leucovorin) and FOLFIRI (irinotecan, fluorouracil pyrimidine and leucovorin) in pts with advanced GC, and the total response rates (RRs) were 13%, 27% and 40%, respectively; the time to progression (TTP) values were 3.2 m, 4.9 m and 6.0 m, respectively; the OS were 6.8 m, 9.5 m and 11.0 m, respectively [10]. Based on available data, FOLFIRI is a promising option for pts with advanced GC [9–11].

This open, randomized, phase II, two-center study was designed to determine whether there is an optimal chemotherapy regimen for advances GC between the FOLFIRI and FOLFOX from the perspective of efficacy and safety.

## RESULTS

### Baseline patient characteristics

**Figure 1:**
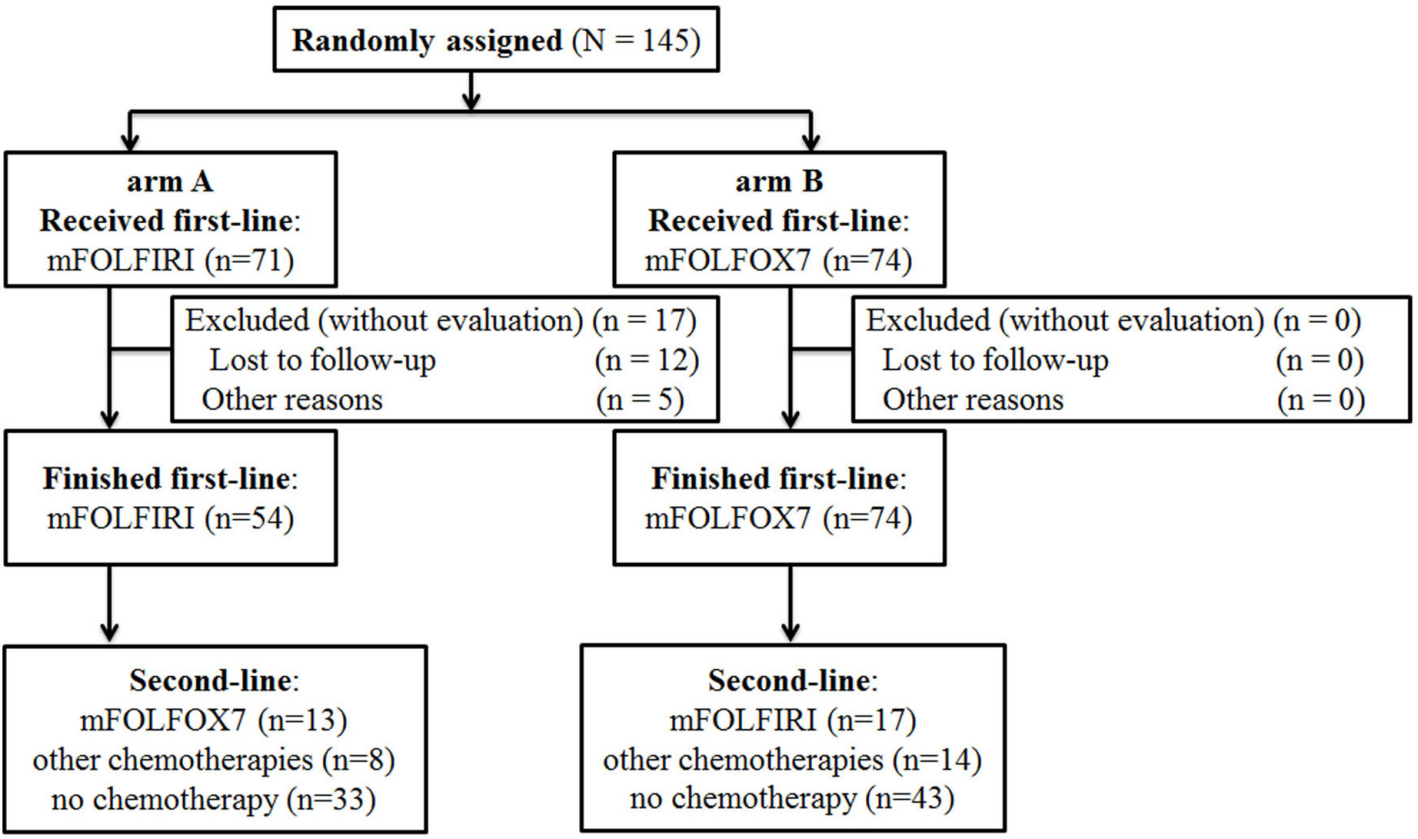
Consort diagram Consort of included patients. mFOLFOX7(modified leucovorin, fluorouracil, and oxaliplatin), mFOLFIRI (leucovorin, fluorouracil, and irinotecan).

Because the pt enrollment was slow, recruitment was stopped in September 2012. From April 2008 to September 2012, 145 pts (71 in arm A; 74 in arm B) were included at West China Hospital, Sichuan University, and Sichuan Cancer Hospital. Seventeen pts were considered late dropouts mainly due to refusal of treatment. As a result, full analysis set (FAS) consisted of 128 pts (54 in arm A; 74 in arm B)(Figure 1). As a result, the assessable population consisted of 128 pts(54 in arm A; 74 in arm B), of which in the second-line treatment 13 pts received mFOLFOX7, 17 pts mFOLFIRI, 22 pts other regimens, such as paclitaxel, capecitabine, etoposide and so on, and the remaining 76 pts with no treatment after first-line chemotherapy (Supplementary Table 1). No one pursued targeted therapy in non-chemotherapy group in the study duration except one patient in the arm B took part in a clinical trial of everolimus.

In the whole population, 104 pts were male, 41 pts were female. The median age was 52.6 (range, 25.0–80.0) years old. No major imbalances were found between the two arms in terms of the baseline characteristics based on the evaluable population (Table 1). The cutoff date for the survival data was October 2013, with a median potential follow-up time for the entire cohort of 9.5 m (range, 0.5 to 42.3 m).

**Table 1:** Patient characteristics

Parameter	ArmA: mFOLFIRI	Arm B:mFOLFOX7
Demographic characteristics		
No. of patients	71	74
Male	71%	73%
Female	29%	27%
Age, years		
Median	53	52
Range	25–80	26–79
ECOG performance status		
0	17%	13%
1	35%	40%
2	48%	47%
Primary tumor resected Metastatic disease	69%	66%
Metastatic site	92%	91%
Liver only	7.4%	4.1%
Liver included	38.9%	24.3%
Liver excluded	53.7%	71.6%
Adjuvant chemotherapy		
Yes	70.4%	83.8%
No	29.6%	16.2%
Signet ring cell included		
Yes	83.3%	85.1%
No	16.7%	14.9%
Degree of differentiation		
Low	44.4%	44.6%
Middle	9.3%	12.2%
Other	46.3%%	43.2%

Abbreviations: mFOLFIRI: folinic acid, fluorouracil, and irinotecan; mFOLFOX7: folinic acid, fluorouracil, and oxaliplatin; ECOG: Eastern Cooperative Oncology Group.

### Treatment exposure

Pts in both arms received a median of 4 cycles (range, 1 to 10) of treatment for first-line chemotherapy. During the entire therapy, there was no therapy-related death. The median dose-intensity was no less than 85%, which was similar in both treatment arms. Additionally, dose reduction was reported in 14 cases in arm A and 6 cases in arm B. For pts who received second-line treatment, the median treatment cycles were 3 cycles (range, 1 to 10) for arm A and 2 cycles (range, 1 to 7) for arm B.

### Efficacy of progression-free survival

**Figure 2:**
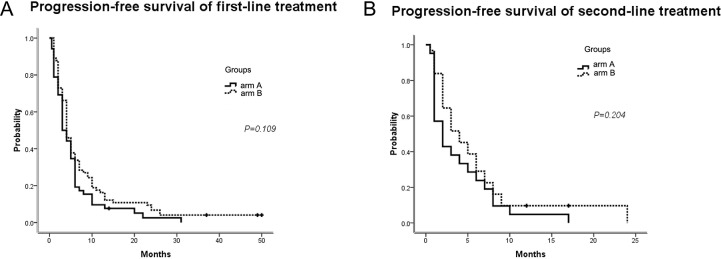
PFS of first-line and second-line treatments (**A**) Median PFS for the first-line treatment; (**B**) Median PFS for the second-line treatment. PFS, progression-free survival; arm A: mFOLFIRI; arm B: mFOLFOX7.

According to the Kaplan-Meier analysis, the median PFS for the first-line treatment was 2.9 m (95% *CI*, 1.9 to 4.1 m) for arm A (mFOLFIRI) versus 4.1 m (95% *CI*, 3.2 to 4.8 m) for arm B (mFOLFOX7; *p* = 0.109; Figure 2). In the second-line treatment, the PFS was 2.0 m (95% *CI*, 0.5 to 3.5 m) for arm A compared with 4.2 m (95% *CI*, 2.0 to 6.0 m) for arm B (*p* = 0.204; Figure 2B). According to the results, the pts treated with mFOLFOX7 first obtained a longer PFS benefit for the entire treatment. Additionally, the Cox regression model using an enter selection approach suggested the two independent prognostic factors for improved first-line PFS were a high degree of differentiation histologically (*p* = 0.006) and a greater number of chemotherapy cycles (*p* = 0.0001).

### Efficacy of overall survival

**Figure 3:**
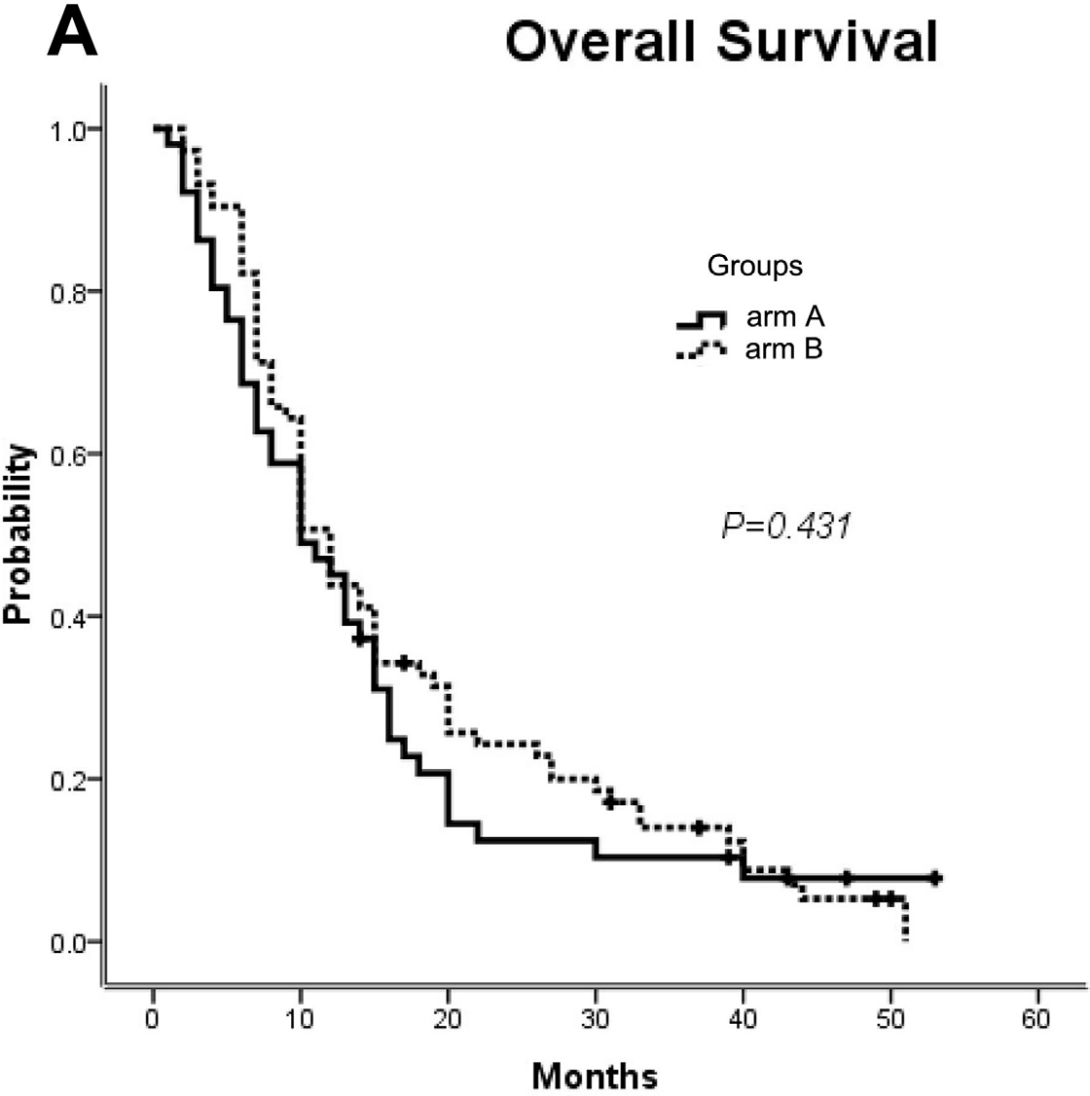
OS for all of the patients Median OS for arm A versus arm B. OS, overall survival; mFOLFOX7(modified leucovorin, fluorouracil, and oxaliplatin), mFOLFIRI (leucovorin, fluorouracil, and irinotecan); arm A: mFOLFIRI; arm B: mFOLFOX7.

Based on the available data, the median OS was 9.9 m (95% CI, 6.0 to 13.5 m) for arm A versus 12.0 months for arm B (95% CI, 10.3 to 13.7 m; *p* = 0.431; Figure 3). Similarly, two independent prognostic factors for improved OS were no dose reduction of first-line chemotherapy drugs (*p* = 0.055) and a shorter interval time between progression on first-line chemotherapy and the first cycle of second-line treatment (*p* = 0.028).

### Efficacy of the disease control rate

Only one CR was observed with arm A (1.9%) versus two with arm B (2.7%). The RRs were 11.2% with arm A compared with 9.5% with arm B, while the DCRs were 59.3% and 66.3% for arm A and arm B, respectively, with no statistical significance(*p* = 0.850). Based on the multinomial logistic regression analysis, only two independent prognostic factors were found to be significant for response: age (*p* = 0.0001) and the number of cycles of chemotherapy (*p* = 0.005).

### Efficacy of mFOLFIRI/mFOLFOX7 VS. mFOLFOX7/mFOLFIRI per protocol set (PP)

**Figure 4:**
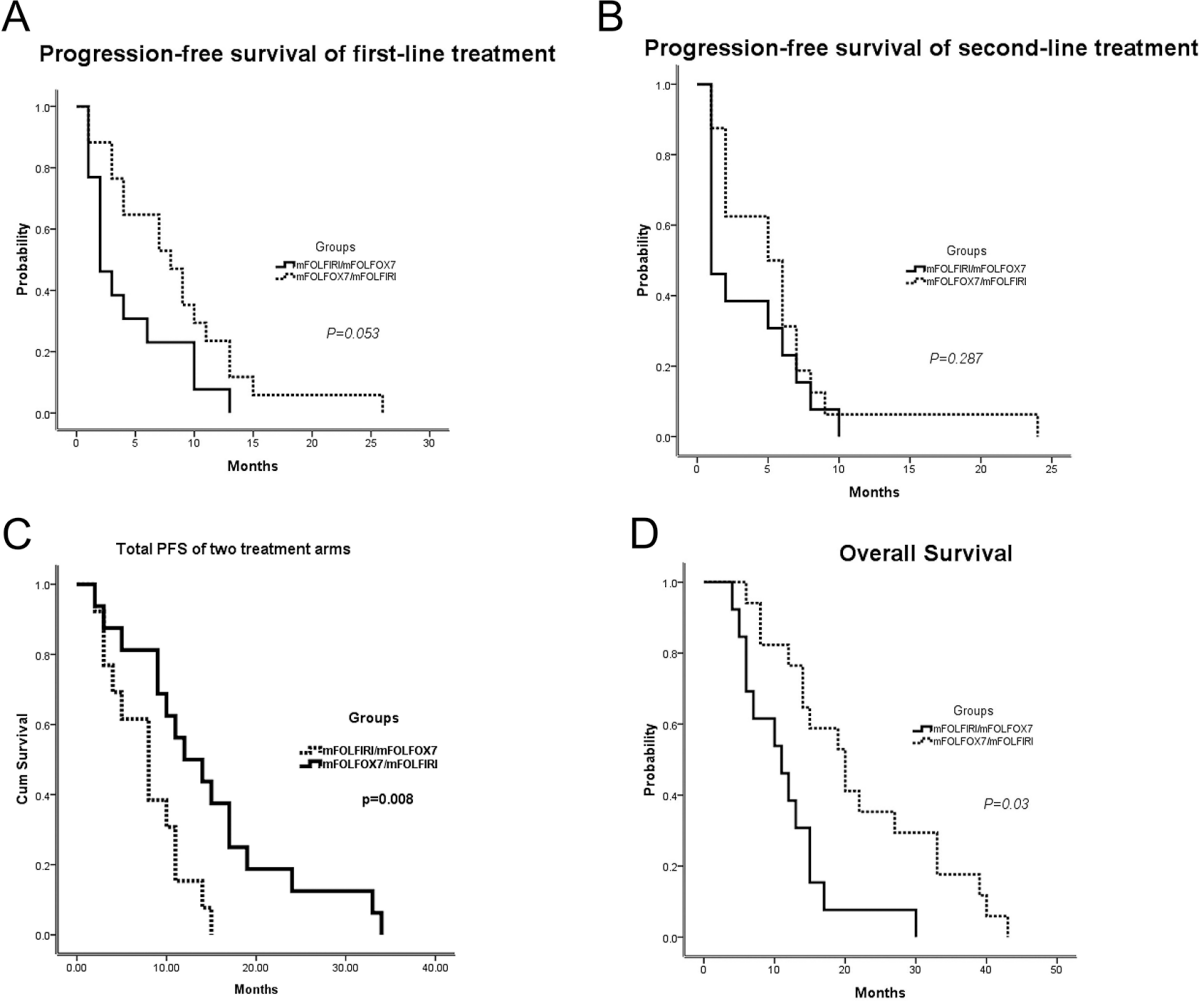
Survival outcomes of patients who completed treatments with mFOLFIRI followed by mFOLFOX7 or the reverse sequence Thirteen patients in arm A and 17 in arm B finished the sequential therapies as the protocols were analyzed. (**A**) Median PFS for the first-line treatment; (**B**) Median PFS for the second-line treatment; (**C**) The total PFS for the combination of first-line and second-line. (**D**) Median OS for both populations. PFS, progression-free survival; OS, overall survival; mFOLFOX7(modified leucovorin, fluorouracil, and oxaliplatin), mFOLFIRI (leucovorin, fluorouracil, and irinotecan).

Notably, however, only 13 pts in arm A and 17 in arm B completed treatment with mFOLFIRI followed by mFOLFOX7 or the reverse sequence as the protocol recommend. The median PFS for the first-line treatment was 2.1 m (95% *CI*, 0.6 to 3.4 m) for the mFOLFIRI/mFOLFOX7arm versus 8.0 m (95% *CI*, 4.0 to 12.0 m) for the mFOLFOX7/mFOLFIRI arm (*p* = 0.053; Figure 4A). Additionally, the median PFS values for the second-line treatment were 1.2 m for the mFOLFIRI/mFOLFOX7arm versus 5.1 m (95% *CI*, 1.9 to 8.1 m) for the mFOLFOX7/mFOLFIRI arm (*p* = 0.287; Figure 4B). Total PFS was 8.1m (95% CI, 4.6–11.4 m) for the mFOLFIRI/mFOLFOX7 group compared with 12.2 m (95% CI, 6.1–17.9 m) for the mFOLFOX7/mFOLFIRI group (*p* = 0.008; Figure 4C).

Surprisingly, the difference in the median OS between the two groups was statistically significant: 20.2 m in the mFOLFOX7/mFOLFIRI arm (95% *CI*, 13.4 to 26.6 m) compared with 11.0 m in them FOLFIRI/mFOLFOX7 arm (95% *CI*, 5.1 to 16.9 m; *p* = 0.03; Figure 4D). Indeed, it was provocative that the sequence of mFOLFOX7/mFOLFIRI PP population had double the median OS of the reverse sequence arm. In order to explore any potential caveats with this observation, baseline characteristics were analyzed between13 pts in arm A and 17 pts in arm B. And no major imbalances were found between the two arms (Supplementary Table 2). Among them, performance status and age were near the statistically significant edge, which might be the factors leading to a longer OS for mFOLFOX7/mFOLFIRI sequence.

The independent prognostic factors for OS improvement were a high degree of differentiation (*p* = 0.028), no dose reduction of first-line chemotherapy drugs (*p* = 0.034), a first-line response (*p* = 0.016) and no second-line chemotherapy delay (*p* = 0.005).

### Toxicity

All of the pts were available for the adverse event analysis. The treatments were well tolerated in both arms. National Cancer Institute CTCAE grade 3–4 neutropenia (34%) and grade 3 sensory neuropathy (12%) were more frequent with arm B. However, pts in arm A had more grade 3 delayed diarrhea (6%) and grade 2 alopecia (45%). Other frequently reported adverse events were predominantly grade 1/2, including thrombocytopenia, anemia, nausea, anorexia, fatigue, stomachache, mucositis, and liver function abnormalities, without a difference between the two arms (Table 3). Additionally, 32% of pts in arm A and 34% in arm B underwent chemotherapy delay because of toxicity. Despite the dose reduction, both regimens were well-tolerated with acceptable and manageable toxicities in the treatment.

**Table 2:** Disease control rates of the two arms

Event Rates	Arm A: mFOLFIRI (*n* = 54)		Arm B: mFOLFOX7 (*n* = 74)	
	No.	%	No.	%
**Disease control rate**	32	59.3	49	66.3
**Complete response**	1	1.9	2	2.7
**Partial response**	5	9.3	5	6.8
**Stable disease**	26	48.1	42	56.8
**Progression disease**	17	31.5	18	24.3
**Not assessable**	5	9.3	7	9.5

Abbreviations: mFOLFIRI: folinic acid, fluorouracil, and irinotecan; mFOLFOX7: folinic acid, fluorouracil, and oxaliplatin.^*^p = 0.021.

**Table 3:** Frequency of toxicities (percentage)

Toxicity	Arm A: mFOLFIRI (*n* = 71)				Arm B: mFOLFOX7 (*n* = 74)			
	G1	G2	G3	G4	G1	G2	G3	G4
**Neutropenia**	5. 6	10	21	4	0	15	27	7
**Sensory neuropathy**	0	0	0	0	1.4	0	12	0
**Delayed diarrhea**	2.8	5.6	6.0	0	1.4	0	1.0	0
**Nausea**	5.6	0	5.6	0	2.8	0	2.8	0
**Vomiting**	5.6	0	5.6	0	2.8	0	2.8	0
**Alopecia**	0	13	0	0	0	45	0	0
**Hand-foot syndrome**	0	0	0	0	0	0	0	0
**Thrombocytopenia**	0	0	5.6	0	0	0	2.8	0

Abbreviations: mFOLFIRI: folinic acid, fluorouracil, and irinotecan; mFOLFOX7: folinic acid, fluorouracil, and oxaliplatin; G, Grade.

## DISCUSSION

Notably, the prognosis of GC has been poor, although progress has been made in new therapeutic treatments and development of early diagnosis, and the 5-year survival rate remains less than 20% [4]. As a result, it is urgent to choose a better treatment combination as well as the best sequence among the available therapeutic strategies and to optimize the OS of advanced GC pts and/or the quality of life.

What are the future directions in the palliative chemotherapy treatment of advanced gastric cancer? Notably, CF was the basic treatment of gastric cancer. Because of the cisplatin-related adverse events and efficacy of capecitabine, the substitution of FOLFOX has been one of the most widely used regimens in the first-line therapy of GC with a considerable advantage [12]. Meanwhile, based on the V306 results, FOLFIRI also shows great advantage in the treatment of gastric cancer [9]. Similarly, a recent study published in *Journal of Clinical Oncology* was designed to compare the efficacy of ECX and FOLFIRI in the first-line treatment of GC, and the regimens of second-line were predefined (FOLFIRI for the ECX group and ECX for the FOLFIRI group) [13]. Additionally, the outcome indicated that FOLFIRI was an acceptable regimen in the first-line treatment of GC. Finally, what about advanced GC treated with FOLFOX or FOLFIRI in the first-line setting?

In April 2008, we initiated the first randomized study of FOLFIRI versus FOLFOX7 in Chinese pts with advanced GC. Based on the available data, the median PFS of arm A (mFOLFIRI) was 2.9 m (95% CI, 1.9 to 4.1 m) versus 4.1 m (95% CI, 3.2 to 4.8 m) for arm B (mFOLFOX7). Although the differences were not significant, the pts treated with mFOLFOX7 first obtained a PFS benefit trend from the whole treatment. Additionally, the median OS was 9.9 m (95% CI, 6.0 to 13.5 m) for arm A versus 12.0 m for arm B (95% CI, 10.3 to 13.7 m). Notably, pts treated with mFOLFOX7 followed by mFOLFIRI per protocol benefited from a longer OS than those who received mFOLFIRI/ mFOLFOX7. The DCR values were 59.3% and 66.3% for arm A and arm B, respectively, the result of which was not significant (*p* = 0.850).

Thereafter, in 2010, the preliminary results of a similar study in Korea suggested that the median survival was 11.3 m in 40 pts treated with mFOLFOX4 followed by mFOLFIRI compared with 9.7 m in 37 pts treated with mFOLFIRI followed by mFOLFOX4 (*P* = 0.143); the median second-line time to progression(TTP) was 6.4 m versus 5.7 m (*P* = 0.015). mFOLFOX4 demonstrated a 37.5% RR and a 2.9 m median TTP compared with mFOLFIRI, which demonstrated a RR of 27% and a TTP of 2.9 min the first-line therapy (*P* = 0.154). In the second-line setting, mFOLFOX4 showed a RR of 10.8% and a TTP of 1.7 m, while mFOLFIRI achieved an RR of 15.4% and a TTP of 2.2 months (*p* = 0.036). The conclusion was reached that both sequences had a similar efficacy in OS; however, mFOLFOX4 followed by mFOLFIRI was slightly better in TTP, a result that was consistent with the subgroup analysis of our study [14].

Using the PP analysis of pts who completed treatment with mFOLFIRI followed by mFOLFOX7 or the reverse sequence in our study, the median PFS for the first-line treatment was 2.1 m for the mFOLFIRI/mFOLFOX7 arm versus 8.0 m for the mFOLFOX7/mFOLFIRI arm (*P* = 0.053). Additionally, the median PFS values for the second-line treatment were 1.2 m for arm A versus 5.1 m for arm B (*P* = 0.287). Total PFS was 8.1m for the mFOLFIRI/mFOLFOX7 group compared with 12.2m for the mFOLFOX7/mFOLFIRI group (*p* = 0.008). Besides, the difference in the median OS between the two groups was statistically significant; the OS for the mFOLFOX7/mFOLFIRI arm was 20.2 m, while that of the mFOLFIRI/mFOLFOX7 arm was 11.0 m (*P* = 0.03). Therefore, as a sequential treatment strategy, the higher absolute advantage of mFOLFOX7/mFOLFIRI was significant compared with pts who received other sequences or those without second-line chemotherapy. Until recently, a systematic review showed that second-line therapy largely decreased the death risk by 18%, and chemotherapy could reach a reduction of approximately 27%, particularly with the addition of remucirumab [15]. Hence, we believe our conclusions about the roles of mFOLFOX7/mFOLFIRI in the palliative treatment of advanced GC pts make sense.

However, based on the Cox regression analysis, we believe that, regarding the absolute benefit of palliative chemotherapy, a high degree of differentiation, the number of cycles of chemotherapy, and no dose reduction of chemotherapy drugs play important roles in the whole treatment. Additionally, if regimens are well-tolerated with acceptable and manageable toxicities, sufficient drug dose intensity and treatment cycles should be given to the pts.

However, there were still some limitations in this study. Above all, the final analyzed sample size of this prospective trial was relatively small due to the limited eligible pts. Because the current research was not foundation supported but was launched by investigators, early dropout and lost follow-up of the pts were apparent. Limited sample size, which was much less than the population in the protocol, resulted in a shortage of statistical power. Furthermore, a substantial proportion of pts did not obtain second-line therapy because of lack of money, family disagreement or self-unwilling. Although limited data were available to evaluate the characteristics of these pts, it was a true reflection of the cancer treatment situation in clinical practice. As a result, the choice of first-line therapy is particularly important for the whole treatment. Third, information regarding the QOL was not evaluated, a finding that could be calculated from the treatment adverse events. To accurately evaluate the chemotherapy influence on QOL, large phase III studies are needed.

To the best of our knowledge, this is the first prospective trial with full data concerning the choice of an optimal chemotherapy regimen in the first-line treatment of advanced GC with FOLFOX or FOLFIRI. With the advantages and disadvantages listed above, our results indicate that both regimens achieve similar efficacy; however, mFOLFOX7 followed by mFOLFIRI seems to have better clinical outcomes. Hence, further phase III studies are warranted to confirm this difference and to develop the standard care for advanced gastric cancer.

## MATERIALS AND METHODS

### Patient eligibility and exclusion criteria

Previously untreated pts aged between 18 and 75 years with an Eastern Cooperative Oncology Group (ECOG) performance status of 0–2 and life expectancy > 4 months were eligible if they had histologically proven gastric or gastroesophageal adenocarcinoma with at least one site of unidimensionally measurable disease(RECIST), adequate bone marrow function (hemoglobin, ≥ 90 g/L; absolute neutrophil count, ≥ 2.0 × 10^9^ cells/L; platelet count, ≥ 100 × 10^9^ cells/L), hepatic function (alkaline phosphatase, ≤ 3 upper limits of normal (UNL); total bilirubin, ≤ 1.5 UNL; AST and ALT, ≤ 3UNL) and renal function (creatinine, ≤ 135 mmol/L). Previous adjuvant chemotherapy, if given, must have been completed at least 6 months before inclusion. Pts with central nervous system metastases, current diarrhea ≥ grade 2, symptomatic angina pectoris, disease confined to previous radiation fields, second malignancies or bowel obstruction were excluded from the study.

The treatment protocol was approved by the medical ethics committee of West China Hospital, Sichuan University and Sichuan Cancer Hospital (Clinical trial information: ChiCTR-TRC-08000167). Signed informed consent was required before all of the eligible pts were enrolled. Additionally, the study was performed in accordance with the ethical standards put forth in the 1964 Declaration of Helsinki.

### Chemotherapy

Pts were randomized (1:1) according to the following regimen: a 2-hour infusion of folinic acid 200 mg/m^2^ followed by a 46-hour infusion of 5-FU 2,400 mg/m^2^ every 2 weeks, either with irinotecan 150 mg/m^2^ (mFOLFIRI, arm A) or with oxaliplatin 85 mg/m^2^(mFOLFOX7, arm B) as a 2-hour infusion on day 1, repeated every 2 weeks. The pts received first-line chemotherapy until progression or unacceptable toxicity. The second-line treatment was predefined (mFOLFOX7 for arm A and mFOLFIRI for arm B).

Pts with intolerance of toxicity related to oxaliplatin or irinotecan and no progressive disease during the first-line treatment could receive folinic acid and 5-FU alone until disease progression. Paclitaxel alone or in combination with cisplatin, capecitabine or 5-FU was recommended after the failure of second-line treatment, as described previously [16].

Based on the most severe toxicity during the last treatment, the drug decreased to 75% of the original dose for the first adjustment and to 50% of the original dose for the second time. Dose modification of single drug or components of the regimen was performed corresponding to the expected toxicity from that agent. Specifically, the dose of 5-FU was reduced for any related toxicity exceeding the National Cancer Institute Common Terminology Criteria for Adverse Events (NCI-CTCAE, v3.0) grade 2, such as diarrhea, mucositis, neutropenia or thrombocytopenia. Irinotecan dose modifications were performed if related grade 3–4 toxicity occurred, including neutropenia, thrombocytopenia, and diarrhea. Oxaliplatin dose was modified to 75% in case of grade 2 paresthesia, and if persistent, to 50%. And oxaliplatin should be omitted from the regimen if persistent painful paresthesia or grade 3 neurotoxicity.

Treatment was discontinued for pts with more than two dose adjustments. At the beginning of each cycle, the treatment was suspended when the neutrophil count was less than 1.5 × l0^9^/L and/or platelets were less than 75 × l0^9^/L and/or non-hematologic toxicity was above grade 2. Additionally, the whole treatment was ceased if a delay from the start of the next 2 week cycle was longer than 2 weeks.

### Evaluation criteria

Physical examination, blood count measurement, hepatic and renal function testing and measurement of the levels of tumor markers were carried out every cycle. Toxicity evaluations were based on the NCI - CTCAE, which were assessed before each 2-week treatment [17]. Radiological evaluations of measurable lesions were conducted at baseline and were repeated every two courses using contrast-enhanced computed tomography or magnetic resonance imaging [18]. The evaluation of tumor response was assessed according to RECIST criteria. The reviews of all radiological scans were performed by two independent radiologists.

The primary endpoint was progression-free survival (PFS) defined as the time duration from randomization until progressive disease (PD) after chemotherapy as well as death from any cause. For every individual patient, the total PFS was the sum of first-line and second-line PFS time. Secondary endpoints were OS calculated from random assignment to death resulting from any cause or the date of the last follow-up, at which point the data were censored. Disease control rates (DCRs, including complete response, partial response, and stable disease), response rates (RRs, total number of complete response, and partial response) and safety evaluations were also collected.

### Statistical strategies

Randomization was performed using a minimization technique, stratifying pts by treatment center [19]. The planned sample size was 100 in each arm, considering the two-sided log-rank test to have 80% power to detect a 20% difference in the proportion of pts without progression at 6 months. Baseline information was assessed by Student's *t*-test and chi-square test. The OS and PFS curves were estimated by the Kaplan-Meier method, and the comparison of the curves was analyzed using the log-rank test [20]. Multivariate analysis of the prognostic factors for survival outcomes was performed using the Cox regression model and an enter selection approach, and multinomial logistic regression analysis was used to evaluate the effect factors for DCR [21]. Age, histological differentiation, cycles of chemotherapy, dose reduction of chemotherapy agents, interval time between the first-line and second-line chemotherapy were included in the analysis.

### Human rights statement and informed consent

All procedures followed were in accordance with the ethical standards of the responsible committee on human experimentation (West China Hospital, Sichuan University and Sichuan Cancer Hospital, P.R. China) and with the Helsinki Declaration of 1964 and later versions. Informed consent or substitute for it was obtained from all patients for being included in the study.

## SUPPLEMENTARY MATERIALS TABLES



## Abbreviations

GC: gastric cancer
mFOLFIRI: folinic acid, fluorouracil and irinotecan
mFOLFOX7: folinic acid, fluorouracil and oxaliplatin
OS: overall survival
DCR: disease control rate
PFS: progression-free survival
QOL: quality of life
pts: patients
m: months
5-FU: 5-Fluorouracil
ECF: epirubicin, cisplatin and 5-FU
EOF: epirubicin, oxaliplatin and 5-FU
ECX: epirubicin, oxaliplatin and capecitabine
IF: irinotecan and 5-FU
LF: fluorouracil and leucovorin
CLF: cisplatin, fluorouracil and leucovorin
RRs: response rates
TTP: time to progression
CTCAE: Common Terminology Criteria for Adverse Events
PD: progressive disease
ECOG: Eastern Cooperative Oncology Group
FAS: full analysis set
