# Supplementary material for: Prospective randomized phase II study of FOLFIRI versus FOLFOX7 in advanced gastric adenocarcinoma: a Chinese Western Cooperative Gastrointestinal Oncology Group Study
Source: Oncotarget. 2017 Jun 9;8(58):97890–9. doi: 10.18632/oncotarget.18426 (PMC5716699; doi:10.18632/oncotarget.18426)
Supplement: Supplementary file 1 [file oncotarget-08-97890-s001.pdf]

Prospective randomized phase II study of FOLFIRI versus FOLFOX7 in advanced gastric adenocarcinoma: a chinese western cooperative gastrointestinal oncology group study

Supplementary Materials

Supplementary Table 1: Second-line treatments

Treatment	arm A (54)	arm B (74)
None	33	43
mFOLFOX7	13	0
mFOLFIRI	0	17
Docetaxel+Cisplatin	1	0
Oxliplatin+ S1	1	0
Docetaxel+S1	1	0
Irinotecan+Paclitaxel liposome	1	0
Docetaxel + mFOLFIRI	1	0
Docetaxel+ Capecitabine	1	0
Etoposide+Leucovorin+Fluorouracil+Cisplatin	1	0
Taxol+ Cisplatin+ Fluorouracil	1	0
Irinotecan+ Cisplatin	0	2
Taxol+ Fluorouracil	0	3
Taxol	0	2
Taxol+ Cisplatin	0	3
S1	0	3
Everolimus clinical trial	0	1

Abbreviations: mFOLFIRI: folinic acid, fluorouracil, and irinotecan; mFOLFOX7: folinic acid, fluorouracil, and oxaliplatin

Supplementary Table 2: Patient characteristics

Parameter	mFOLFIRI/mFOLFOX7	mFOLFOX7/mFOLFIRI	*P* value
Demographic characteristics			
No. of patients	13	17	
Male	9	11	0.554
Female	4	6	
Age, years			
Median	58 ± 5.8	52 ± 9.0	0.065
ECOG performance status			
0	0	4	0.056
1	8	10	
2	5	3	
Primary tumor resected Metastatic disease	9	8	0.242
Metastatic site	11	17	0.262
Liver only	2	0	0.181
Liver included	3	4	
Liver excluded	8	13	
Adjuvant chemotherapy			
Yes	2	1	0.397
No	11	16	
Signet ring cell included			
Yes	1	2	1.000
No	12	15	
Degree of differentiation			
Low	6	9	0.534
Middle	1	2	
Other	6	6	

Abbreviations: mFOLFIRI: folinic acid, fluorouracil, and irinotecan; mFOLFOX7: folinic acid, fluorouracil, and oxaliplatin; ECOG: Eastern Cooperative Oncology Group.
